# Supplementary material for: Identification of Freezing-Responsive microRNAs and Their Targets in Chinese Jujube by Small RNA and Degradome Sequencing
Source: Int J Mol Sci. 2026 Sep 4;27(17):7912. doi: 10.3390/ijms27177912 (PMC13566890; doi:10.3390/ijms27177912)
Supplement: Supplementary file 1 [file ijms-27-07912-s001.zip › Supplementary Material S2/novel_miR_190.pdf]

Provisional ID	:	novel_miR_190
Score total	:	1.1
Score for star read(s)	:	-1.3
Score for read counts	:	0
Score for mfe	:	0.8
Score for randfold	:	1.6
Score for cons. seed	:	
Total read count	:	283
Mature read count	:	257
Loop read count	:	26
Star read count	:	0

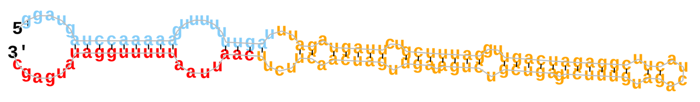

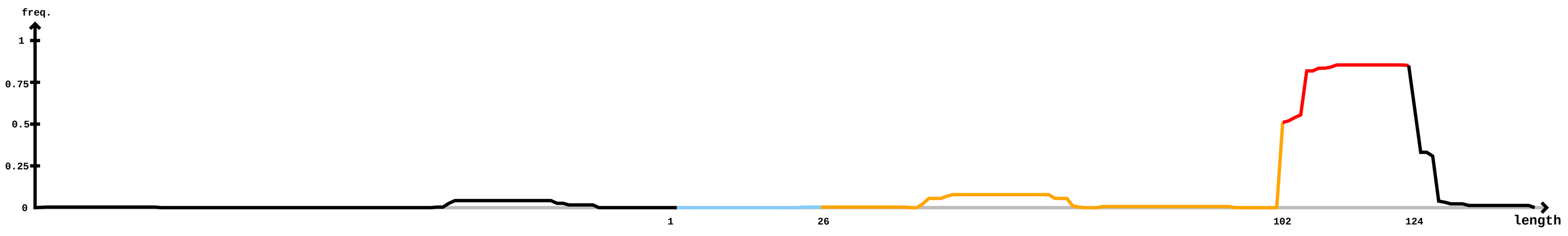

Star Mature

```
5'- cugucaaagauuccugaugagucagagauucuuuuguugauuagucuuuaaucagaacccagaucauaugggucuugacuguccugauagucggcccaucuuguggaugauccaaaaaguuuuuuugauuuagaugauucugcuuuagguugacuagaggcuucaucagauguuucugagucgucugauaguugaucaacuucuucaauuaauuuuuggauaugagcaagcauuucuuucuuugaucug -3'
    .....(((((.((((((....)))).)).)))))...(((((((.....)))))))....(((((((.(((((((..((((((....)))))))))))))(((((..(((.((((((((......((((...((.(((((..(((((((..(((((((((((.((....)).)))))).))))).)))).))).))))).))...))))....)))))))))))..))))).............)))))))
```

read	reads	mm	sample
cugucaaagauuccugauC	1	1	JCK-3
cauaugggucuugacugucUug	1	1	LT-3
uaugggucuugacugucA	5	1	LT-3
uaugggucuugacugucUug	1	1	JCK-1
uaugggucuugacugucUug	1	1	JCK-2
augggucuugacugucUugauagu	1	1	LT-3
augggucuugacugucUugauagu	4	1	JCK-3
ugauGuagaugauucugc	1	1	LT-2
uCgguugacuagaggcuucauc	2	1	JCK-2
uCgguugacuagaggcuucauc	5	1	JCK-3
Cgguugacuagaggcuucaucaga	6	1	JCK-2
Cgguugacuagaggcuucaucaga	1	1	JCK-3
Cgguugacuagaggcuucaucaga	3	1	JCK-1
uugacuagaggcuucaucaga	1	0	JCK-3
uugacuagaggcuucaucaga	2	0	JCK-2
uugacuagaAgcuucaucaga	1	1	JCK-3
ugacuagaggcuucaucagau	2	0	JCK-2
ugacuagaggcuucaucagaug	1	0	JCK-1
cugaAucgucugauaguugauc	1	1	JCK-1
cugaAucgucugauaguugauc	1	1	JCK-2
caauuaauuuuuggauaugagc	17	0	JCK-1
caauuaauuuuuggauaugagc	10	0	LT-2
caauuaaCuuuuggauaugagc	1	1	LT-3
caauuaauuuuuggauaugagc	16	0	LCK-1
caauuaauuuuuggauaugagc	30	0	JCK-3
caauuaauuuuuggauaugagc	9	0	LT-3
caauuaauuuuuggauaugagc	47	0	JCK-2
caauuaauuuuuggauaugagc	16	0	LCK-2
caauuaauuuuuggauaugagc	2	0	LT-1
caauuaauuuuuggauaugagc	3	0	LCK-3
caauuaauuuuuggauaugagca	2	0	LCK-3
caauuaauuuuuggauaugagca	1	0	LT-1
caauuaauuuuuggauaugagca	3	0	JCK-2
aauuaauuuuuggauaugagc	2	0	JCK-1

```
                                                                                                                                                                                                   Star                                                                                    Mature
cugucaaagauuccugaugagucagagauucuuuuguugauuagucuuuaaucagaacccagaucauaugggucuugacuguccugauagucggcccaucuuguggaugauccaaaaaguuuuuuugauuuagaugauucugcuuuagguugacuagaggcuucaucagauguuucugagucgucugauaguugaucaacuucuucaauuaauuuuuggauaugagcaagcauuucuuucuuugaucug
..............................................................................................................................................................................................................aauuaauuuuuggauaugagc......................    1    0    JCK-3
...............................................................................................................................................................................................................auuaauuuuuggauaugag.......................    4    0    JCK-2
...............................................................................................................................................................................................................auuaauuuuuggauaugag.......................    1    0    JCK-1
...............................................................................................................................................................................................................auuaauuuuuggauaugagcaagc..................    1    0    LT-3
................................................................................................................................................................................................................uuaauuuuuggauaugagcaag...................    1    0    JCK-1
................................................................................................................................................................................................................uuaauuuuuggauaugagcaag...................    2    0    JCK-3
................................................................................................................................................................................................................uuaauuuuuggauaugagcaag...................    2    0    JCK-2
.................................................................................................................................................................................................................uaauuuuuggauaugagcaag...................    1    0    JCK-2
.................................................................................................................................................................................................................uaauuuuuggauaugagcaag...................    1    0    JCK-3
.................................................................................................................................................................................................................uaauuuuuggauaugagcaagc..................    1    0    LT-3
.................................................................................................................................................................................................................uaauuuuuggauaugagcaagc..................    14   0    JCK-2
.................................................................................................................................................................................................................uaauuuuuggauaugagcaagc..................    25   0    LCK-2
.................................................................................................................................................................................................................uaauuuuuggauaugagcaagc..................    18   0    LCK-1
.................................................................................................................................................................................................................uaauuuuuggauaugagcaagc..................    8    0    JCK-3
.................................................................................................................................................................................................................uaauuuuuggauaugagcaagc..................    11   0    JCK-1
.................................................................................................................................................................................................................uaauuuuuggauaugagcaagc..................    2    0    LT-1
...................................................................................................................................................................................................................auuuuuggauaugagcaagc..................    2    0    LT-3
...................................................................................................................................................................................................................auuuuuggauaugagcaagc..................    1    0    JCK-1
...................................................................................................................................................................................................................auuuuuggauaugagcaagcau................    1    0    JCK-1
...................................................................................................................................................................................................................auuuuuggauaugagcaagcau................    1    0    JCK-3
.....................................................................................................................................................................................................................uuuuggauaugagcaagca.................    2    0    LT-3
......................................................................................................................................................................................................................uuuggauaugagcaagcau................    1    0    LT-3
......................................................................................................................................................................................................................uuuggauaugagcaagcauuuc.............    1    0    JCK-1
......................................................................................................................................................................................................................uuuggauaugagcaagcauuuc.............    1    0    JCK-3
......................................................................................................................................................................................................................uuuggauaugagcaagcauuuc.............    1    0    JCK-2
..................................................................................................................................................................................................................................caagcauuucuuucuuugauc..    2    0    JCK-3
..................................................................................................................................................................................................................................caagcauuucuuucuuugauc..    1    0    JCK-1
..................................................................................................................................................................................................................................caagcauuucuuucuuugauc..    1    0    JCK-2
```
